# Morphological characteristics and clinical significance of the distal femur in patients with hemophilia-related knee arthritis

**DOI:** 10.1097/MD.0000000000022986

**Published:** 2020-11-20

**Authors:** Qiang Gao, Yunfeng Yao, Juehua Jing

**Affiliations:** Department of Orthopaedic Surgery, Second Affiliated Hospital of Anhui Medical University, Heifei, China.

**Keywords:** clinical significance, distal femur, hemophilia-related knee arthritis, morphological characteristics, total knee arthroplasty

## Abstract

This retrospective study aimed to define the morphological characteristics of the distal femur in patients with hemophilia-related knee arthritis (HA) and develop precise femoral component installation during total knee arthroplasty (TKA) using a reference axis.

Computed tomography (CT) was performed in 75 patients [HA group: 34 patients, 48 knees; osteoarthritis (OA group): 41 patients, 48 knees] during 2017–2019. CT scans were constructed into three-dimensional models. We measured the medial (MPC) and lateral (LPC) posterior condyle widths, lateral anteroposterior (LAP) height, medial anteroposterior (MAP) height, mediolateral epicondyle (ML) width, and depths of the anterior patellar groove (X2) and the intercondylar notch (X4). Also, angles were measured between the posterior condylar line (PCL) and surgical transepicondylar axis (STEA) (PCA angle), anteroposterior axis (APA angle) and STEA (APSA angle), anterior condylar line (ACL) and STEA (ACA angle), and clinical transepicondylar axis (CTEA) and PCL (CTA angle). ML/MAP, ML/LAP, X4/LAP, X2/LAP, and LPC/ML ratios were calculated.

There were no significant differences in any angles between the HA and OA groups (*P* > .05). However, the HA group had a smaller MPC (*P* < .05) and larger X4 than the OA group (*P* < .05). ML, ML/LAP, X2, MAP, and LAP showed no significant differences between the 2 groups.

ML, ML/LAP, and PCA showed no significant differences between the 2 groups. During TKA in hemophilia-related knee arthritis patients, the femoral component can be installed with PCL as the reference axis, although individual differences should be considered.

## 1 Introduction

Hemophilia is an inherited, X-chromosome-linked, recessive bleeding disorder with a reported incidence of hemophilia A in 1/5000 male neonates ^[1]^ and hemophilia B in 1/30,000 male neonates.^[2]^ Hemophilic arthritis, provoked by recurrent articular bleeding, is a common complication of severe hemophilia. In most cases, bleeding episodes begin during early childhood and continue into adult life. Because children do not have a conscious sense of self-protection during this period, motor organ injury with bleeding into the joint is quite common.^[3]^ The most important musculoskeletal bleeding site is the joint space.^[4]^ The exact musculoskeletal bleeding mechanisms have not been fully elucidated, it is probably that hemosiderosis in the synovium induces an inflammatory response that causes stimulates angiogenesis and immune system activation.^[5]^ Over 90% of bleeding episodes occurs within the musculoskeletal system, 80% occurs within the joints.^[4]^

**Figure 1:**
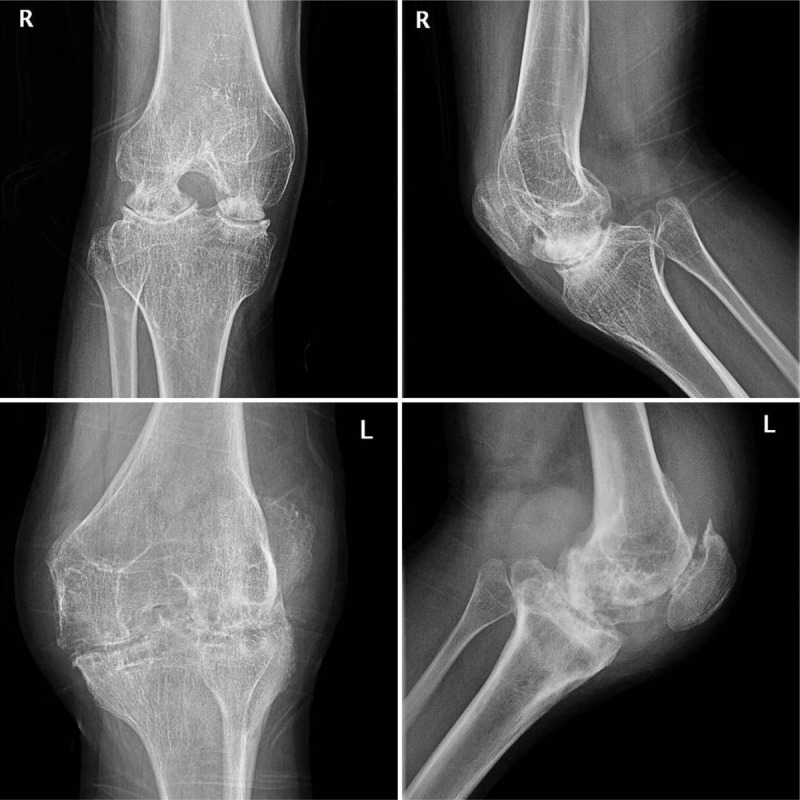
Radiography of a patient with hemophilic knee arthritis in both knees.

Some studies have shown characteristic radiographic changes due to hemophilia-related knee arthritis. Repeated bleeding into the knee joint leads to asymmetrical development and widening of the epiphysis, deepening of intercondylar notch, asymmetrical development of internal and external femoral condyle, femoral condyledevelopmental deformity, narrowing of the joint gap, formation of subchondral cysts, an irregular subchondral surface, and osteoporosis (Fig. 1).^[6]^ Due to the femoral condyle developmental deformity, whether the femoral component can be installed in HA using PCL as reference axis in the same way as in OA need deeply thought.

TKA is an effective method for treating advanced hemophilic knee arthritis. To date, however, the morphological characteristics of the distal femur have not been studied.^[7]^ Recurrent articular bleeding causes distal femoral developmental deformity, which leads to difficulty selecting the correct rotational alignment of the femoral component and the installation position during TKA. No relevant studies have been reported on how to refer to the distal femoral anatomical landmarks during TKA of a patient with hemophilic knee arthritis. Therefore, we studied the morphological characteristics of the distal femur in patients with hemophilic knee arthritis who required TKA, with the aim of determining the best technique for selecting the correct rotational alignment of the femoral component and the most advantageous installation position.

## 2 Materials and methods

All patients with TKA in our hospital from July 2017 to December 2019 were enrolled in this study. There were 34 patients (48 knees) with hemophilic knee arthritis and 541 (620 knees) with osteoarthritis (OA), among whom 296 fulfilled the OA inclusion criteria. Among the 296 patients, in chronological order, 48 knees (41 patients) were extracted using systematic sampling. The composite of the 2 groups was divided into a study group (HA group: 34 patients, 48 knees) and a control group (OA group: 41 patients, 48 knees). Prior to undergoing TKA, we collected computed tomography (CT) scans to determine the morphological characteristics of the distal femur of the 96 knees in 75 patients. The average age of HA group was 34.5 ± 10.1 years (range 20–50 years), the average age of the OA group was 61.5 ± 2.7 years (range 55–65 years). The Ethics Committee of our hospital has approved the study (Batch Number: PJ-YX2020-010).

Inclusion criteria: male sex; no severe knee varus or valgus; for the HA group, age range of 20 to 50 years and for the OA group 55 to 65 years; body mass index 22 to 25 kg/m^2^; absence of factor VIII inhibitor in HA group. Exclusion criteria: presence of a knee tumor; prior revision knee surgery; traumatic knee arthritis; rheumatoid knee arthritis; previous knee infection; previous knee surgery; or osteotomy. All surgery was completed by the same senior orthopedic surgeon.

Morphological characteristics of the distal femur were determined in all 75 patients (96 knees) using a Siemens Definition AS40 multi-slice spiral CT device (Siemens, Erlangen, Germany). For this procedure, the patients were positioned so they were lying supine with their knee joints stretched straight. The scanning was performed parallel to the horizontal plane of the articular surface of the medial and lateral condyle of the distal femur. The scanning range was >10 cm from the superior pole of the patella to the distal tibial plateau, and the scanning interval was set at 2 mm. Three-dimensional reconstruction of scanned images was carried out using computer system reconstruction software, and the scanned raw data were burned onto an optical disc and exported to MIMICS 10.01 (Materialise, Leuven, Belgium). The measurement was carried out through the horizontal plane of the medial and lateral femoral epicondyles.

**Figure 2:**
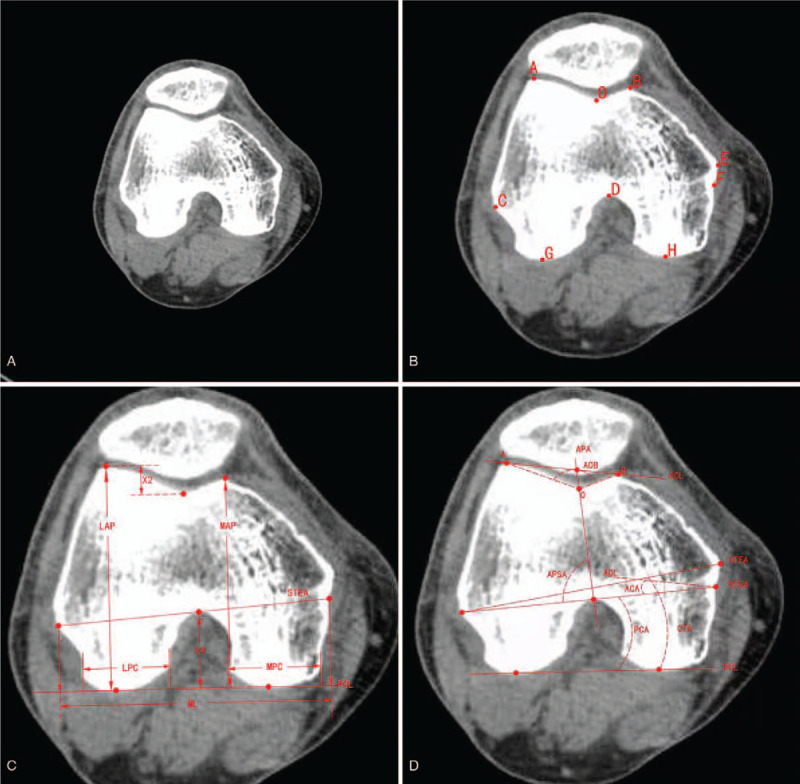
Measurement parameters for the distal femur. A Computed tomography (CT) image of the distal femur. B Location of landmarks on an axial CT image. *A* Highest point of the lateral condyle. *B* Highest point of the medial condyle. *C* Most prominent point of the lateral epicondyle. *D* Top of the intercondylar notch. *E* Most prominent point of the medial epicondyle. *F* Most concave point of the medial epicondyle. *G* Most prominent point of the lateral posterior condyle. *H* Most prominent point of the medial posterior condyle. *O* Most distal point at the bottom of the trochlear groove. C Morphological parameters of the distal femur. *LAP* vertical distance from the highest point of the external epicondyle of the femur to the PCL, *MAP* vertical distance from the highest point of the medial epicondyle of the femur to the PCL, *LPC* the lateral posterior condyle width, *MPC* the medioposterior condyle width, *X2* the depth of the anterior patellar groove, *X4* the intercondylar notch, *ML* the most concave point of the distal medial femoral condyle and the most prominent point of the external condyle, the distance after the vertical projection of the connection line at the PCL. D Rotational axis and angle of the distal femur. *ACA* angle between ACL and STEA, *PCA* angle between PCL and STEA, *APSA* angle between APA and STEA, *CTA* angle between PCL and CTEA.

The MPC, LPC, LAP, MAP, ML, X2, and X4 were measured (Fig. 2A, 2B, 2C). The STEA, CTEA, PCL, ACL, and APA were identified on the CT images (Fig. 2D). The PCA, APSA, CTA, and ACA angles were measured, and the ML/MAP, ML/LAP, X4/LAP, X2/LAP, MPC/ML, and LPC/ML ratios were calculated. Three independent orthopedic surgeons evaluated all the data to ensure objectivity and rule out interobserver bias.

### 2.1 Statistical analysis

All statistical analyses were performed using SPSS 16.0 statistical software (IBM, Inc, Armonk, NY, USA). Both sample populations were normally distributed. Metrological data subject to approximate normal distribution were presented as means ± standard deviations. The HA and OA groups were analyzed using an independent sample *t* test. A value of *P* < .05 was considered to indicate a significant difference.

## 3 Results

The morphological parameters for the distal femur in the 2 groups are shown in Table 1. There was no significant difference between the HA and OA groups (*P* > .05) for the ML width, LAP height, MAP height, LPC width, or X2 depth. There was a between-group difference, however, in the MPC and X4 measurements: The HA group had a smaller MPC (*P* < .05) and a larger intercondylar notch (*P* < .05) than the OA group.

**Table 1:** The morphological parameters of the distal femur.

Morphological parameters	HA group(n = 34) Mean ± SD (range)	OA group (n = 41) Mean ± SD (range)	t	*P* values
ML width (mm)	82.97 ± 5.79 (73.7, 93.1)	82.67 ± 3.68 (77.4, 91.6)	0.214	.831
LAP heigtht (mm)	61.76 ± 5.30 (52.1, 71)	63.29 ± 3.38 (56.9, 69.8)	−1.192	.241
MAP height (mm)	58.35 ± 5.87 (46.5, 70.2)	60.30 ± 3.66 (53.2, 67.7)	−1.382	.175
MPC width (mm)	24.03 ± 3.94 (13.2, 28.9)	27.75 ± 3.58 (23.4, 38.2)	−3.432	.001
LPC width (mm)	27.60 ± 4.92 (19, 39.2)	27.50 ± 2.20 (23.4, 32.7)	0.095	.925
X4 depth (mm)	26.71 ± 5.64 (17.9, 39.9)	21.29 ± 3.10 (14.3, 26.3)	4.128	.000
X2 depth (mm)	8.75 ± 3.92 (2.2, 16)	8.07 ± 2.74 (2.8, 13.4)	0.691	.493

The proportional results of relevant data for the distal femur are presented in Table 2. The X4/LAP ratio was higher in the HA group than in the OA group (*P* < .05), whereas there was no significant difference between the groups for the X2/LAP ratio (*P* > .05). The ML/MAP, ML/LAP, and LPC/ML ratios also were not significantly different between the 2 groups (*P* > .05). The MPC/ML ratio, however, was lower in the HA group than in the OA group (*P* < .05).

**Table 2:** The proportions of relevant data of the distal femur.

Proportions	HA group(n = 34)Mean ± SD (range)	OA group(n = 41) Mean ± SD (range)	t	*P* values
ML/LAP	1.35 ± 0.11 (1.12, 1.55)	1.31 ± 0.08 (1.20, 1.50)	1.432	.159
ML/MAP	1.43 ± 0.12 (1.23, 1.70)	1.38 ± 0.09 (1.18, 1.55)	1.798	.079
MPC/ML	0.29 ± 0.05 (017, 0.35)	0.34 ± 0.04 (0.27, 0.46)	−3.843	.000
LPC/ML	0.33 ± 0.06 (0.25, 0.49)	0.33 ± 0.03 (0.30, 0.39)	0.031	.976
X4/LAP	0.43 ± 0.08 (0.29, 0.57)	0.35 ± 0.04 (0.24, 0.43)	4.920	.000
X2/LAP	0.14 ± 0.07 (0.04, 0.29)	0.13 ± 0.04 (0.05, 0.20)	1.019	.315

The PCL was 4.3°± 3.0° internally rotated relative to the STEA in the HA group and 3.3°± 2.4° in the OA group. There was no significant difference in the PCA between the 2 groups (*P* > .05). The condylar twist angle was not significantly different in the 2 groups (*P* > .05). The APSA was 88.2°±10.7° in the OA group and 83.4°±6.4° in the HA group (*P* > .05). There was no significant difference in the ACA angles between the 2 groups (Table 3).

**Table 3:** The relevant angles of the distal femur.

Angles	HA group(n = 34) Mean ± SD (range)	OA group(n = 41) Mean ± SD (range)	t	*P* values
ACA	6.3°± 4.1°(1°, 17°)	6.2°± 4.5°(0°, 15°)	0.101	.920
PCA	4.3°± 3.0°(1°, 12.4°)	3.3°±2.4°(1°, 10°)	1.187	.241
CTA	4.8°± 3.0°(0°, 10.4°)	5.5°± 3.0°(1°, 12.1°)	−0.801	.427
APSA	88.2 ± 10.7(76°, 111°)	83.4 ± 6.4(71°, 96.3°)	1.892	.066
AOB	135.6°± 19.1°(101°, 162°)	139.1°± 8.9°(118.2°, 156°)	−0.819	.419

## 4 Discussion

Recurrent intraarticular bleeding associated with hemophilic knee arthritis can lead to irreversible lesions in the articular structure. It can cause synovial hypertrophy, synovitis, degenerative changes in the articular cartilage, and subcartilage bone tissue destruction—ultimately resulting in joint deformity, fibrosis, and limited movement during adulthood.^[8]^ Hemophilic arthropathy can affect one or more joints—primarily the knee, hip, ankle, and elbow joints—among which the knee joint is most commonly affected. If serious, patients can completely lose their ability to work and walk.^[9]^ Although timely administration of the coagulation factor can avoid secondary joint lesions, 80% patients with severe hemophilia in developing countries suffer complications with joint lesions because of the price of drugs and the effects of their medical conditions.^[10]^

In this study, we found that there was no significant difference in the morphological parameters of the distal femur except for X4 and MPC. The X4 and the X4/LAP ratio was higher in the HA group than in the OA group (*P* < .05). Wangroongsub et al^[11]^ thought that the proper entry point for the femoral intramedullary guiding rod during TKA should be 1.5 ± 2.01 mm medial and 12.0 ± 2.72 mm superior to the top of the femoral intercondylar notch. In addition, the MPC and MPC/ML ratio were lower in the HA group than in the OA group (*P* < .05). The MPC and LPC of HA group were (24.03 ± 3.94) mm and (27.60 ± 4.92) mm, the MPC and LPC of OA group were (27.75 ± 3.58) mm and LPC (27.50 ± 2.20) mm, it is not difficult to find that the asymmetrical development of internal and external femoral condyle in HA group.

TKA is an effective method for treating advanced hemophilic arthritis. The success of TKA depends on precise placement of the implant, balance between the ligaments, and recovery of limb alignment.^[12]^ The rotation alignment of the femoral component after TKA greatly influences the mechanics of motion, function of the knee joint, and survival of the prosthesis.^[13]^ It can also maintain the rectangular shape of the flexion space of the knee joint and the smoothness of the joint. Rotational malalignment of the femoral component in TKA is associated with poor outcomes, including flexion instability, patellar maltracking, premature wear of the polyethylene, stiffness, anterior knee pain, and patient dissatisfaction.^[13,14]^ During TKA, the rotation alignment and installation position of the femoral component are generally based on the anatomical signs of the distal femur. The most widely used anatomical signs are the APA, STEA, and PCL.

The transepicondylar axis may be divided into the STEA and CTEA. The STEA was established by drawing a line from the most prominent point of the lateral femoral epicondyle to a sulcus on the medial epicondyle, and the CTEA was established by drawing a line from the most prominent part of the lateral femoral epicondyle to the most prominent part of the medial epicondyle. The STEA is widely seen as a gold standard for rotational alignment of the femoral component.^[15]^ It can define the flexion gap of the rectangle, and some believe that the rotational direction of the femoral component is parallel to the STEA. Franceschini et al^[16]^ suggested that, when the knee bends to 90°, the STEA is perpendicular to the femoral mechanical axis and the tibial mechanical axis. But STEA has a fatal weakness that is not visible intraoperatively. In the present study, the CTA and ACA were not significantly different in the HA and OA groups (*P* > .05).

The APA, from the highest point in the intercondylar notch to the deepest point of the trochlea, has an important role in the reference axis of the rotating alignment of the femoral component. In 1 study, the distal femur was evaluated in 50 cadavers, with the results showing that the Whiteside line is perpendicular to the STEA.^[17]^ Cho et al^[18]^ showed that the APA can be applied to posterior condylar bone erosion or hypoplasia, but it is difficult to identify varus or valgus deformity due to destructive arthritis or trochlear dysplasia in the knee. Using APA alone to determine the rotation of the femoral component in patients with medial OA may lead to excessive external rotation.^[19]^

The PCL was determined by drawing a line between the most distal point of the distal lateral to the distal medial femur internally rotated 3° relative to the STEA. In most cases, determining the STEA is difficult in most cases, and the PCL is much easier to determine intraoperatively: The anterior bone cut is usually made in 3° of external rotation relative to the PCL, and the STEA is parallel to the 3° of external rotation of the PCL.^[20]^ Some studies have shown that in most TKAs, the PCL is much easier to find intraoperatively, with 3° to 5° external rotation of the PCL typically used as a reference axis to install the femoral component to balance the flexion gap. Nevertheless, one must consider the individualization factor.^[21]^ We generally use PCL as a reference axis when installing the femoral component. Combined with the postoperative conditions of 34 patients in the HA group, the femoral component was installed with the PCL as the reference axis, the force line of the lower limbs returned to normal after the operation, and the knee joint movement was obviously improved compared with the previous. From 1 to 3 years after the operation, the prosthesis was in place without any discomfort. Therefore, PCL is perfect as a reference axis for HA.

There was no significant difference in the ML, ML/LAP, or PCA between the HA and OA groups (*P* > .05). When TKA was performed, the reference axis between the 2 groups showed no significant difference. Consequently, in patients with hemophilic knee arthritis, the femoral component can be installed using the same reference axis as that used for TKA in the presence of OA. Again, however, individual differences should be considered.

There are several limitations of this study. There was a large age difference between the 2 groups, the number of cases was small, and it was a retrospective study. Thus, a larger sample size and a better index are needed. Although there is a large age difference between the 2 groups, the degree of the knee joint damage in the 2 groups was roughly the same, indicating that patients with hemophilic knee arthritis, with its earlier onset and presentation with knee joint lesions at young-adult ages reach the same level of severity as that seen in older patients with OA.

## 5 Conclusions

There were no significant differences in the PCA, ML, or ML/LAP ratio between the HA and OA groups. Consequently, when TKA is performed in patients with hemophilic knee arthritis, the femoral component can be installed with PCL as the reference axis, as it is for OA patients. Individual differences, however, should always be considered.

## Acknowledgments

We would like to thank all the participants in the studies.

## Author contributions

**Conceptualization:** Yunfeng Yao.

**Data curation:** Qiang Gao.

**Formal analysis:** Qiang Gao.

**Funding acquisition:** Juehua Jing.

**Investigation:** Qiang Gao.

**Resources:** Yunfeng Yao.

**Writing – original draft:** Qiang Gao.

**Writing – review & editing:** Qiang Gao, Yunfeng Yao.
